# The Influence of cis-Regulatory Elements on DNA Methylation Fidelity

**DOI:** 10.1371/journal.pone.0032928

**Published:** 2012-03-06

**Authors:** Mingxiang Teng, Curt Balch, Yunlong Liu, Meng Li, Tim H. M. Huang, Yadong Wang, Kenneth P. Nephew, Lang Li

**Affiliations:** 1 Harbin Institute of Technology, School of Computer Science and Technology, Harbin, Heilongjiang, China; 2 Department of Medical and Molecular Genetics, Indiana University School of Medicine, Indianapolis, Indiana, United States of America; 3 Center for Computational Biology and Bioinformatics, Indiana University School of Medicine, Indianapolis, Indiana, United States of America; 4 Medical Sciences Program, Indiana University, Bloomington, Indiana, United States of America; 5 Indiana University Melvin and Bren Simon Cancer, Indianapolis, Indiana, United States of America; 6 Department of Molecular Virology, Immunology and Medical Genetics, Comprehensive Cancer Center, The Ohio State University, Columbus, Ohio, United States of America; 7 Departments of Cellular and Integrative Physiology and Obstetrics and Gynecology, Indiana University School of Medicine, Indianapolis, Indiana, United States of America; 8 Indiana Institute of Personalized Medicine, Departments of Cellular and Integrative Physiology and Obstetrics and Gynecology, Indiana University School of Medicine, Indianapolis, Indiana, United States of America; Oregon State University, United States of America

## Abstract

It is now established that, as compared to normal cells, the cancer cell genome has an overall inverse distribution of DNA methylation (“methylome”), *i.e.*, predominant hypomethylation and localized hypermethylation, within “CpG islands” (CGIs). Moreover, although cancer cells have reduced methylation “fidelity” and genomic instability, accurate maintenance of aberrant methylomes that underlie malignant phenotypes remains necessary. However, the mechanism(s) of cancer methylome maintenance remains largely unknown. Here, we assessed CGI methylation patterns propagated over 1, 3, and 5 divisions of A2780 ovarian cancer cells, concurrent with exposure to the DNA cross-linking chemotherapeutic cisplatin, and observed cell generation-successive increases in total hyper- and hypo-methylated CGIs. Empirical Bayesian modeling revealed five distinct modes of methylation propagation: (1) heritable (*i.e.*, unchanged) high- methylation (1186 probe loci in CGI microarray); (2) heritable (*i.e.*, unchanged) low-methylation (286 loci); (3) stochastic hypermethylation (*i.e.*, progressively increased, 243 loci); (4) stochastic hypomethylation (*i.e.*, progressively decreased, 247 loci); and (5) considerable “random” methylation (582 loci). These results support a “stochastic model” of DNA methylation equilibrium deriving from the efficiency of two distinct processes, methylation maintenance and *de novo* methylation. A role for *cis*-regulatory elements in methylation fidelity was also demonstrated by highly significant (p<2.2×10^−5^) enrichment of transcription factor binding sites in CGI probe loci showing heritably high (118 elements) and low (47 elements) methylation, and also in loci demonstrating stochastic hyper-(30 elements) and hypo-(31 elements) methylation. Notably, loci having “random” methylation heritability displayed nearly no enrichment. These results demonstrate an influence of *cis*-regulatory elements on the nonrandom propagation of both strictly heritable and stochastically heritable CGIs.

## Introduction

DNA methylation is a vital vertebrate animal process intimately linked to the proper regulation of gene expression and the preservation of genomic integrity. In non-embryonic cells, DNA methylation predominantly occurs at the C5 position of cytosines 5′ to adjacent guanines (*i.e.*, transpiring within “CpG” dyads) [1], [2]. In concert with other epigenetic (*i.e.*, DNA sequence-external) events, DNA methylation is one of the primary mediators of cellular lineage commitment and tissue-specific specialization during organismal development [3], [4]. In addition to differentiation, DNA methylation also suppresses the expression/transposition of (potentially mutagenic) repetitive elements, maintains heterochromatinization within satellite DNA (thus facilitating genomic stability), and silences the monoalleles of imprinted genes and the inactive X chromosome, within female somatic cells.

Due to its crucial role in the development and maintenance of differentiated phenotypes, DNA methylation patterns must be precisely replicated during DNA synthesis and cell division. DNA methylation is catalyzed by DNA methyltransferases (DNMTs), of which three active isoforms exist in mammals: DNMT-1, DNMT-3a, and DNMT-3b [1], [2], [5]. Of these, the “maintenance” methyltransferase, DNMT1, associates with the DNA replication machinery (“replisome”) during DNA synthesis and (similar to DNA polymerase-1) is believed to utilize the template strand (“hemimethylated,” or already methylated from the previous cell division) to correctly transfer methyl groups to deoxy-cytosines within the nascent DNA molecule [6], [7], [8]. A current model of DNA methylation posits that DNMT1 acts in a processive, stochastic manner, methylating the new DNA strand in cooperation with the *de novo* DNMTs 3a and 3b, resulting in the very accurate (>99.5%) [9], [10] maintenance of regional DNA methylation levels, without necessarily exact copying of specific methylcytosines [6], [9], [11], [12].

While over 80% of all genomic CpG dinucleotides are methylated in somatic cells, distinct CpG-rich regions (“CpG islands,” CGIs), often associated with gene promoters, remain largely (>70%) unmethylated [1], [13], [14], [15]. CGIs frequently colocalize with essential *cis*-acting DNA elements, including origins of replication, regions of nucleosome depletion [15], [16], and our group [17], [18] and others [15], [19], [20], [21] have also demonstrated CGIs are enriched with transcription factor binding sites (TFBSs). How hypomethylated CGIs are “protected” from methylation is not well understood, but in addition to transcription, is believed to involve zinc-finger proteins possessing a CXXC motif for binding unmethylated CpG-rich sequences [16], [22], [23] that modify chromatin/histones into an “open,” transcriptionally permissive conformation [1], [2]. Likewise, “insulator” DNA-binding proteins have been shown to protect specific active transcriptional modules from DNA methylation and other gene-repressive chromatin modifications [24].

Another possible mechanism of “protecting” CGIs from DNA methylation is via transcription factor recruitment of gene-activating chromatin modifiers (*e.g.*, coactivators such as histone acetyltransferases) that also facilitate decondensation of chromatin [1]. Furthermore, in some active genes, the transcriptional machinery may sterically obstruct promoter-associated CGIs from repressive, DNMT-containing complexes, and our group [25] and others [13], [15], [20], [21] have also shown that silencing of some transcription factors may result in promoter DNA methylation of their downstream “target” genes. However, a large number of silenced genes also remain methylation-free, demonstrating that transcription-associated steric obstruction of DNMTs is but one of several bases for preserving CGI hypomethylation [13], [15]. By contrast, some transcription factors may actually direct gene repression, by recruiting DNMTs 3A and/or 3B for the methylation of specific target genes [26].

Although copying of DNA methylation patterns is very precise in normal cells, the accumulation of methylation errors (*e.g.*, as occurs during aging, various environmental exposures, or dysregulation of chromatin modifiers) can spur the onset of several diseases, including cancer and various developmental, autoimmune, and neurological disorders [2], [27]. Cancer cells in particular, as compared to normal cells, have an overall inverse distribution of genomic DNA methylation, *i.e.*, global hypomethylation and localized hypermethylation (within CGIs) [1], [2]. It has also been demonstrated that cancer cells often possess decreased methylation fidelity, partially associated with increased *de novo* methylation and genomic instability [2], [5], [11], [28], [29], [30], [31]. Nonetheless, despite those many impediments, accurate maintenance of cancer-associated methylation patterns is necessary for the “memory” of gene expression patterns that preserve a tumor-progressive phenotype (thus facilitating growth-advantaged clonal expansion and/or the sustainment of “cancer stem cells”) [23], [32]. Further corroboration for a requirement of DNA methylation maintenance in neoplasia is provided by the finding that DNMT1 knockdown is lethal to transformed cells [33]. However, while a model for DNA methylation fidelity in normal cells is now well supported, the mechanism(s) that preserves tumor-associated methylation patterns, in cancer cells, remains for the most part, unknown.

Based on those previous DNA methylation studies, we determined the DNA methylation levels [34], [35] of over 44,000 CpG-rich oligonucleotides, derived from 12,000 genes, over the course of 1, 3, and 5 cell generations of A2780 ovarian cancer cells. Our overall objective was to examine the fidelity of DNA methylation heritability even in the presence of multiple negative effectors of faithful methylation inheritance, including DNA damage and two cancer cell phenotypes, accelerated cell growth and possible aberrant expression of chromatin-modifying genes [36], [37]. If such fidelity was indeed observed, a secondary objective was to begin to investigate a possible basis(es) for the maintenance of faithful methylation heritability. Those findings demonstrated both strict (unchanged) and stochastic (*i.e.*, fluctuating) methylation heritability patterns over the five cell generations, and the validity of those methylation measurements was also substantiated using multiple, highly accurate normalization procedures. Further, we observed a substantial amount of “random” transgenerational methylation, as might be expected, in DNA-damaged and genetically unstable cancer cells (which already possess the methylation heritability impediments described above) [5], [29], [31], [32], [38]. We also observed that both stochastically and strictly heritable CGIs were highly enriched with transcription factor binding sites (TFBSs), with significant TFBS overlap between parental cell CGI probe loci having similar starting levels of DNA methylation. Randomly propagated CGIs, however, were devoid of such *cis*-regulatory sites, raising the possibility of a biological mechanism(s) that preferentially enforces methylation fidelity within CpG-dense DNA regions having *cis*-regulatory elements, as compared to regions lacking such elements, during the process of tumor progression.

## Results

### Global DNA methylation levels after 1, 3, and 5 cell generations, concordant with cisplatin exposure

We examined transgenerational heritability patterns of faithful DNA methylation maintenance in the ovarian cancer cell line A2780, over the course of 1, 3, and 5 cell divisions, concordant with exposure to a number of variables that might negatively affect DNA methylation fidelity, including DNA damage, accelerated cell division (and thus DNA synthesis), and altered expression of chromatin modifiers.

**Figure 1:**
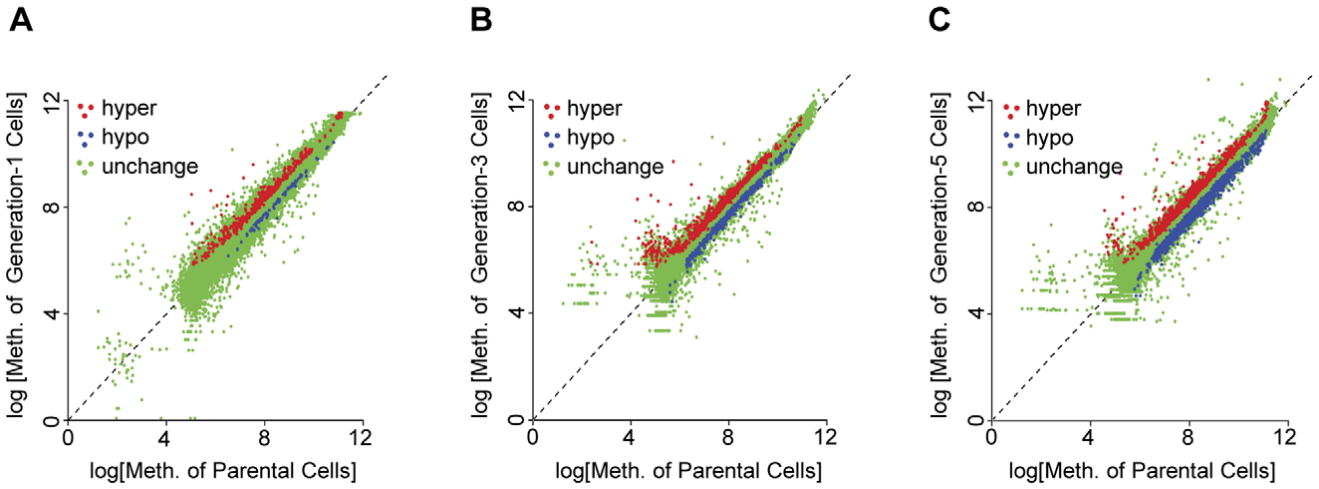
Scatter plots of differential DNA methylation for A2780 cells after 1, 3, and 5 cell generations, coincident with cisplatin DNA crosslinking. The log signals of average observed methylation signals of the parent A2780 cells (x-axis) were compared to the log signals of observed methylation in (**A**) generation-1; (**B**) generation-3, and (**C**) generation-5 cells, respectively. Red represents the hyper methylated CGI probe loci, blue represents the hypo methylated CGI probe loci, and green represents not differentially methylated CGI probe loci.

**Figure 2:**
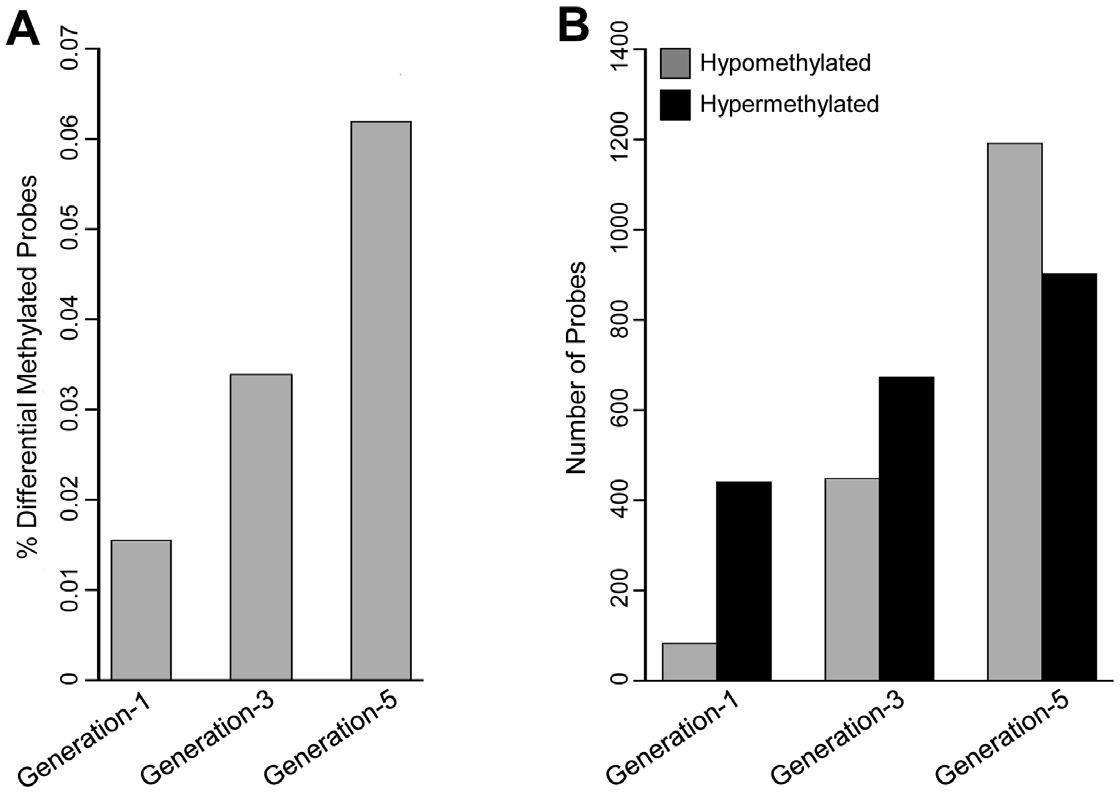
Analysis statistics of differential DNA methylation. (A) Percentages of differentially methylated CGI microarray probes identified by empirical Bayesian model (probability≥0.8) between A2780 cells (baseline) and progeny cell generations 1, 3, and 5 (also treated with the DNA crosslinker cisplatin). Decreased or increased methylation levels from baseline parental cells to progeny cells were defined as the differential methylation; (B) Number of hypomethylated (decreased methylation) and hypermethylated (increased methylation) microarray CGI probes for the cell generations 1, 3, and 5, as compared to the parental cells.

When comparing CGI methylation profiles of first, third, and fifth generation cells, vs. their parental A2780 cells, we observed substantial, progressive increases in both hypermethylated, and hypomethylated CGI probe loci for the three cell generations, coexistent with numerous loci exhibiting no change in DNA methylation (Figures 1 and 2). Despite those considerable changes in DNA methylation, the overall methylation distributions, across generations-1, 3, and 5 were similar (Figure 1), with the methylation status of any specific gene estimated by its probability of differential methylation determined by our previous empirical Bayesian model [39], and a probability threshold of 0.80 (See **Methods**). At that threshold, the proportions of differentially methylated CGI probe loci, as compared to the total number of CGI probe loci, were 0.0154, 0.033, and 0.062 for cell generations 1, 3, and 5, respectively (Figure 2A). We observed progressive and substantial increases in both hypomethylated (85, 448, 1191) and hypermethylated (442, 673, 904) CGI probe loci for generations 1, 3, and 5, respectively (Figure 2B). As shown, 1^st^ generation cells possessed about 49% of the CGI probe loci found hypermethylated in 5^th^ generation cells, but less than 10% of the loci found hypomethylated in 5^th^ generation cells, thus demonstrating a greater rate (about 6 times larger) of hypomethylation than hypermethylation following repeated cell division and DNA damage, similar to previous studies of prostate cancer and other solid tumors [40], [41].

### DNA methylation validation with replicate microarray assessment of generation-3 A2780 daughter cells

**Figure 3:**
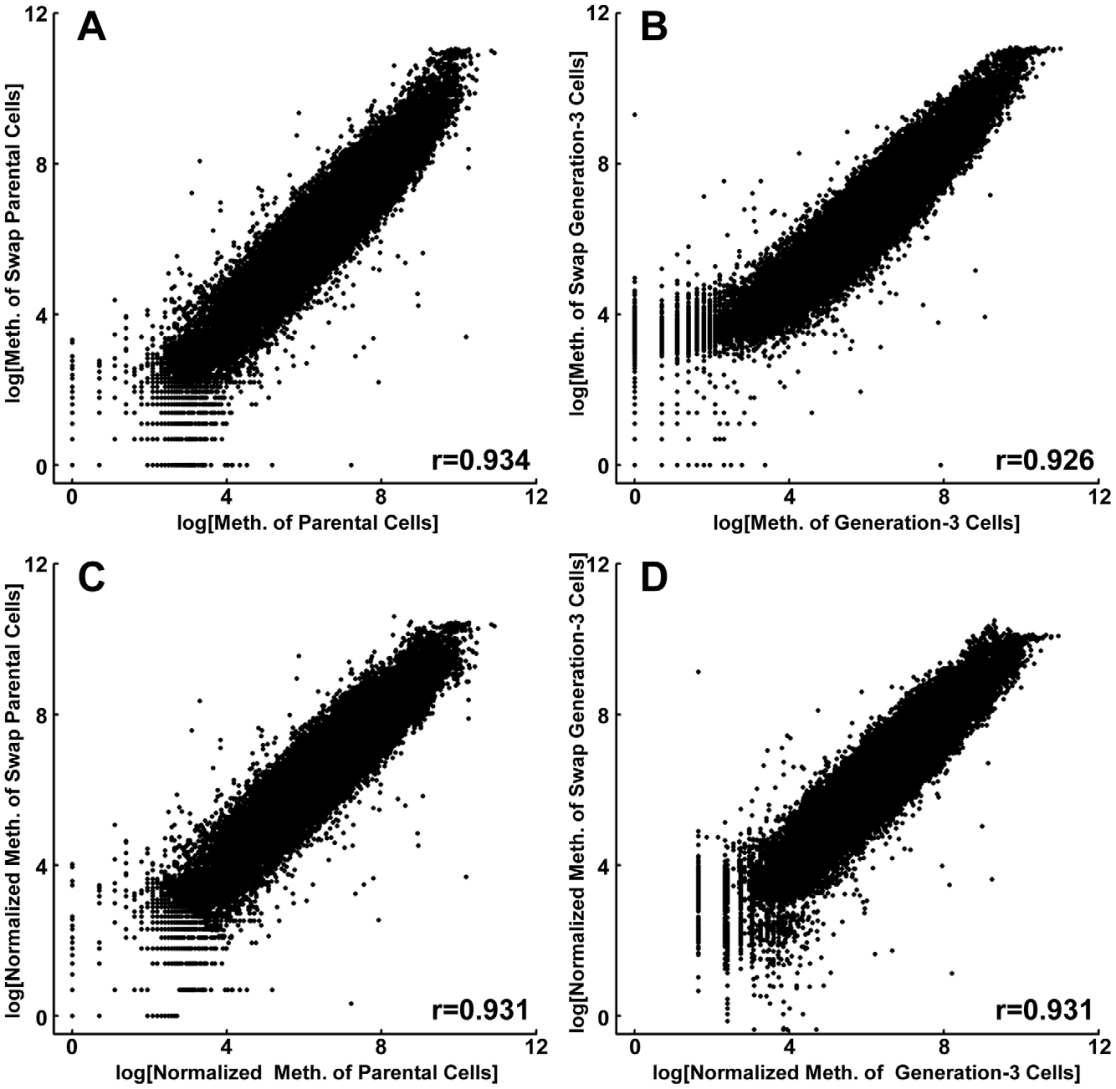
Correlation of DNA methylation intensities between the swap and non-swap microarrays of generation 3 cells. (A) and (B) are scatter plots and correlations of parental and cisplatin treated cells before normalization, respectively. (C) and (D) are scatter plots and correlations of parental and cisplatin treated cells after normalization, respectively.

To substantiate our two-color DNA methylation microarray results, in addition to using standard normalization, we additionally performed a superior technique for validating lack of systematic errors. This control validation entails using the identical experimental procedure, with the sole deviation being a “dye swap,” *i.e.*, using Cy5 (red, in place of Cy3, green) for labeling of the parental cells, and using Cy3 in place of Cy5 for the various generation daughter cells. As shown in Figure 3, the correlation between the swap dyes and non-swap dyes were 0.934 for the generation-3 cells and 0.926 for the parental cells, before Loess normalization, and 0.931 and 0.931 after normalization, respectively. These strong correlations indicate that the methylation signals detected by the microarrays were reproducible, and free of various systematic errors that can confound accurate detection of intensity differences [42]. In the follow-up analysis, we used the average methylation signals of two generation 3 microarray replicates.

### Cell generation-dependent DNA methylation reveals five distinct categories of the propagation of CGI DNA methylation

A current, widely accepted mechanism of DNA methylation maintenance, which has been well validated experimentally [6], [7], [43], is the “stochastic” methylation model, which sets forth that the average methylation levels of specific regions result from the efficiency of two cooperative stochastic processes: heritable maintenance methylation and *de novo* methylation, occurring in concert with DNA replication [8], [11], [12], [44], [45]. In this study, we further subcategorized strictly heritable methylation into two classes, *heritable high* and *heritable low* methylation; and two subclasses showing progressive fluctuation, *stochastic hypermethylation* and *stochastic hypomethylation* (Table 1). In addition, we also observed methylated loci showing neither heritable nor stochastic DNA methylation; those particular loci were categorized into a fifth class, *random methylation*, defined as loci having transgenerational methylation propagation inconsistent with existing (stochastic or heritable) methylation models [11], [12], [44].

**Table 1:** Categorization of CGI probe loci into five distinct classes of DNA methylation propagation.

Categories	Differential Methylation		
	Parental *vs.* Generation-1 Cells	Parental *vs.* Generation-3 Cells	Parental *vs.* Generation-5 Cells
Stochastic Hypomethylation	down	down	down
	even	down	down
	even	even	down
Stochastic Hypermethylation	up	up	up
	even	up	up
	even	even	up
Random Methylation	down	up	down
	down	up	even
	down	even	down
	even	up	down
	even	up	even
	even	down	even
	even	down	up
	up	down	up
	up	down	even
	up	even	up
Heritable Low Methylation	even	even	even
Heritable High Methylation	even	even	even

To determine transgenerational DNA methylation fidelity, methylation levels of specific microarray CGI loci were examined after 1, 3, and 5 cell divisions of the (parental) ovarian cancer cell line A2780. CGI probe loci showing generation-dependent, progressively increased methylation from generations 1 to 5, were defined as the stochastically hypermethylated, while CGI loci showing progressively decreased methylation were considered “stochastically hypomethylated.” Microarray CpG loci demonstrating a mixture of increased and decreased methylation from generations 1 to 5 were considered “randomly methylate,” while CGI loci showing consistently high methylation (>85% of the maximal microarray fluorescence, regardless of generation, also having ≤15% changes between generations) were designated as “heritably highly methylated.” Finally, loci consistently having <15% of the maximal fluorescence (regardless of cell generation, also having ≤15% changes between generations) were categorized as being “heritably lowly methylated.”

**Figure 4:**
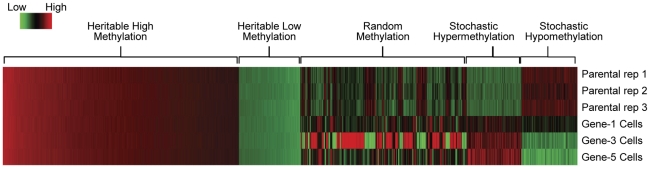
Heatmap and CGI probe loci distributions of the five categories of methylation pattern maintenance. The normalized methylation signals for loci in five different methylation patterns were compared, ranging as vertically Parental replicate dyes and generation 1, 3, 5 cisplatin treated dyes, and horizontally heritable high methylation (1186 CGI loci), heritable low methylation (286 loci), random methylation (582 loci), stochastic hypermethylation (243 loci), and stochastic hypomethylation (247 CGI loci). **“**parental rep 2” indicates the average of parental dyes of non-swap and swap microarray experiments, while “gene-3 cells” is the average of corresponding generation 3 dyes.

Using those five predefined methylation categories (Table 1), we classified generation-dependent methylation propagation patterns of A2780 cell generations (coincident with enhanced resistance to cisplatin) [46], [47]. As shown in Figure 4, the propagation of CGI methylation patterns in progeny (1^st^, 3^rd^, 5^th^ generations) cells, as compared to the parental cells, fell into the five abovementioned categories (also see Table 1 and **Methods**): 1) heritable high methylation (1186 CGI probe loci); 2) heritable low methylation (286 loci); 3) stochastic hypermethylation (243 loci); 4) stochastic hypomethylation (247 loci); and 5) random methylation (defined above, 582 loci) (Figure 4 and Table 2). While these findings are consistent with both the heritable and stochastic models of DNA methylation maintenance [11], [12], they also demonstrate a significant degree of random (*i.e.*, indiscriminate) propagation of CGI loci methylation that might occur following various biological insults [48]. These results, especially random methylation, demonstrate that within any specific cell population, DNA methylation can vary across the genome, in close agreement with a recent study of methylation “entropy” [45].

**Table 2:** TFBS enrichment in CGI probe loci segregating into each of the five methylation propagation categories.

Category	Probe Sequences	Enriched TFBSs Using Background Sequence Set #1^a^(p<2.2*e-5)	Enriched TFBSs Using Background Sequence Set #2^b^(p<2.2*e-5)
Stochastic Hypomethylation	247	31	18
StochasticHypermethylation	243	30	28
RandomMethylation	582	1	0
HeritableLow Methylation	286	47	19
HeritableHigh Methylation	1186	118	115

TFBS frequencies were compared between each of the five sets of probe sequences separately from five methylation categories and the background sequences through Fisher exact test. Two different background sequences sets were used, as described above and in the text, to examine the specificity of TFBS enrichment to the CGI probe loci categorized into the five methylation maintenance categories. The p-value threshold was justified by a factor of 459×5, *i.e.* 0.05/459/5 = 2.2×10^−5^, based on 459 TFBS motifs and 5 comparisons.

a Background Sequence Set #1  =  randomly generated promoter sequences (length/%GC-matched) to the CGI probe loci categorized into the five methylation classes.

b Background Sequence Set #2  =  randomly selected microarray probe sequences (length/%GC-matched) uncategorized into the five methylation classes.

Since our methylation microarray possess over 40,000 60-mer probes, derived from ∼12,000 genes, many genes possessed >1 probes. Consequently, to assess whether probe loci residing within the same CpG island segregated into the same DNA methylation heritability categories, we performed an extended analysis by placing intra-CGI probes into clusters and analyzing the possible DNA methylation heritability categories of probes in each cluster (See **Methods**). Those results showed that, with the exception of probe loci belonging to the “heritable methylation”, the majority of CpG island-colocalized probes did not similarly categorize with one another (data not shown). While this limited analysis precludes any strong inferences from these results, we speculate that the fidelity of DNA methylation could differ between specific subregions within the same CGI, and we could speculate that those better-maintained methylation sequences could also associate with cis-regulatory elements, with such elements not associated to adjacent CGI probe loci.

### Extent of TFBS enrichment in CGIs displaying the five categories of propagation of DNA methylation

Based on previous studies demonstrating CGI are enriched with transcription factor binding sites [14], [15], [18], [19], [20], [49], [50], the stochastic model of methylation inheritance [11], [12], [44], [51], [52], and the necessity of accurate methylation heritability for sustaining cancer phenotypes [23], [32], we assessed the possible influence of *cis*-regulatory DNA elements on DNA methylation fidelity over the course of the five cell generations. Using our published bioinformatics algorithm [18], we assessed TFBS enrichment within CGI probe loci segregating into the five methylation maintenance categories listed in Table 1. To assess the specificity of TFBS enrichment to CGI probe loci, two distinct sets of background sequences were also examined for TFBS motifs, with each set having identical GC content and sequence length (see **Methods**). Both sets of background sequences yielded very similar TFBS enrichment analysis (Table 2), thus validating our background sequence selection (length and GC content-matched). The numbers of enriched TFBSs for CGI probe loci within each DNA methylation category when using randomly generated promoter sequences as background were as follows: heritable highly methylated loci, 118 TFBSs; heritable lowly methylated loci, 47; stochastically hypermethylated loci, 30; and stochastically hypomethylated loci, 31 (again consistent with the hallmark of extensive CGI hypermethylation in cancer cells) [2], [27], [32] (Table 2). Strikingly, we found nearly no TFBS enrichment in CGI probe loci demonstrating random methylation across the three cell generations (Table 2). While this result could have multiple interpretations, one possibility is a reduced stringency of methylation fidelity within CGI probe loci unassociated with *cis*-acting regulatory elements.

**Figure 5:**
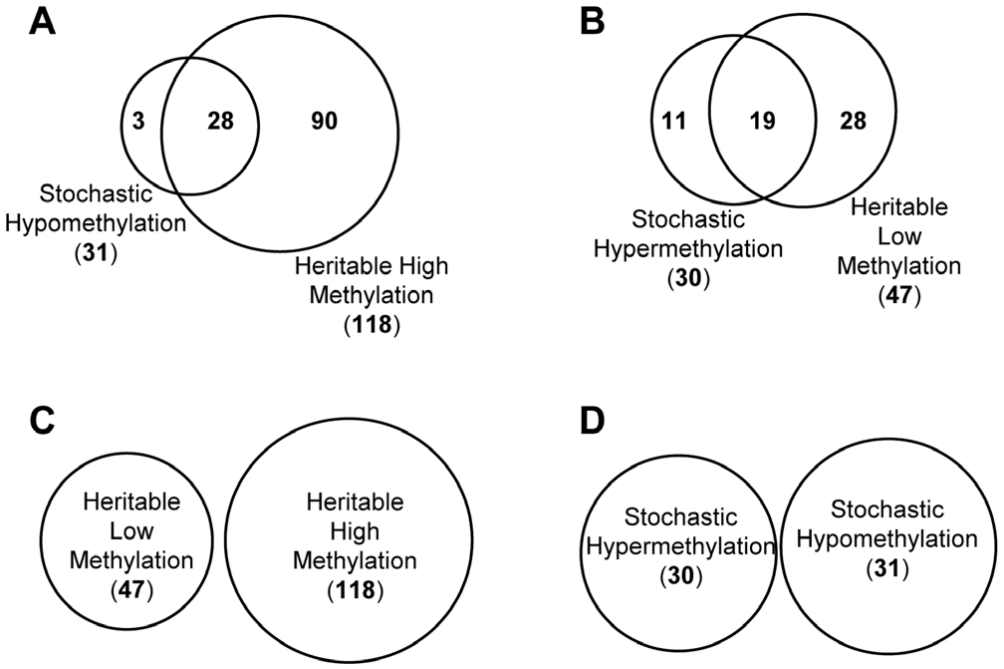
Overlaps of enriched TFBSs among CGI probe loci segregating into the four methylation categories. (**A**) Overlap between enriched TFBSs in stochastically hypomethylated CGI probe loci and in heritably highly methylated CGI probe loci; (**B**) Overlap between enriched TFBSs in stochastically hypermethylated CGI probe loci and heritably lowly methylated CGI probe loci; (**C**) Overlap between enriched TFBSs in heritably lowly methylated CGI probe loci and heritably highly methylated CGI probe loci; and (**D**) Overlap between enriched TFBSs in stochastically hypomethylated CGI probe loci and stochastically hypermethylated CGI probe loci.

Based on prior studies demonstrating CGI association with various essential *cis*-acting DNA elements [13], [15], we next examined the possible influence of TFBSs on the fidelity of CGI propagation. We first compared TFBS enrichment within loci possessing similar initial methylation levels in the parental cells. For example, between TFBSs enriched in stochastically hypomethylated and heritable highly methylated CGI probe loci (*i.e.*, both loci sets being initially significantly methylated in the parental cells), we observed 28 TFBSs in common (Figure 5A). Similarly, 19 of 30 TFBSs enriched in stochastically hypermethylated loci were also present in heritable lowly methylated CGI probe loci, consistent with both sets starting with relatively low methylation (Figure 5B). Likewise, in heritability categories with dissimilar starting levels of methylation in the parental cells, we observed no overlap in TFBS enrichment, *i.e.*, between heritably highly and heritably lowly methylated CGI probe loci, and between stochastically hypomethylated and stochastically hypermethylated CGI probe loci (Figures 5C and 5D). These results are consistent with the stochastic DNA methylation maintenance model, which describes equilibration of DNA methylation, at specific loci, is a function of the methylation efficiencies of methylation maintenance (by DNMT1) and *de novo* methylation, catalyzed by DNMTs,

To further examine the TFBS commonalities between the various overlapping methylation propagation categories, we assessed possible biological functions of their cognate transcription factors (TFs), with specific regard to cancer relatedness and cell cycle progression. TFs corresponding to the TFBSs found enriched within CGIs having steady or high starting methylation levels (*i.e.*, stochastically hypomethylated and heritable highly methylated loci) included TFs associated with tissue-specific differentiation (myogenic factors, including *MYOD1*, *MYOG*, *MYF5*, and *MYF6*), *RAR* family genes (encoding mediators of differentiation of multiple tissue stem cells), oxidative response genes (*NFE2*, *NFE2L2*), and tumor suppressors often downregulated by DNA methylation in various cancers, including *TFAP2A*, *TFAP2B*, and *TFAP2C* (Table 3). While enhanced methylation (and in likelihood, transcription silencing) of these differentiation-associated genes is consistent with the cancer phenotype, we also observed several proto-oncogenic TFs in this group, including *MYC* and *TCF*, *PAX*, and *RUNX* family genes. By contrast, in CGIs with steady or initially low DNA methylation (*i.e.*, stochastically hypermethylated and heritable lowly methylated loci), overlapping TFBSs included those associated with cancer promotion, including various proto-oncogenes such as the *HMG*, *STAT*, and *NKX* families (Table 3), again consistent with the malignant phenotype. However, similar to the enhanced methylation category, we also (paradoxically) observed low methylation of some tumor suppressors, including the *ONECUT* and *FOX* (forkhead) family of TF genes. These results suggest that the maintenance of DNA methylation levels of TFBSs may only partially relate to their associated biological functions (upon binding their cognate TFs), and might also be influenced by the mere presence of specific *cis*-acting elements themselves.

**Table 3:** Enriched transcription factor genes in CGI probe loci within each of the five transgenerational methylation maintenance categories.

Categories	Enriched Transcription Factor Genes
Stochastic Hypermethylation	*FOXD1, FOXF2, FOXJ2, FOXL1, FOXO1, FOXO3, HNF1A, MEF2A, MEF2C, MEF2D, NKX2-5, NKX3-1, NKX6-1, NKX6-2, POU1F1, POU2F1, POU3F2, POU6F1, SOX10, SOX11, SOX12, SOX13, SOX14, SOX15, SOX18, SOX2, SOX21, SOX3, SOX4, SOX5, SOX6, SOX8, SOX9, SRY, STAT1, STAT5A, TBP, ZNF384*
Stochastic Hypomethylation	*ARID5B, ASCL1, HNF4A, HNF4G, LMO2, MYF5, MYF6, MYOD1, MYOG, NFIA, NFIC, NKX2-1, NR2F1, NR2F2, NR5A1, PPARG, REST, SMAD4, TAL1, TCF12, TCF3, TCF4, TFAP2A, TFAP2B, TFAP2C, TFAP4, TFCP2, UBP1, ZBTB6*
Random Methylation	*REST*
Heritable Low Methylation	*AR, ATF2, CDX2, CEBPA, CUX1, FOXD1, FOXO3, FOXO4, HMGA1, HMGA2, HNF1A, IKZF2, LEF1, LHX3, MEF2A, MEF2C, MEF2D, NFIL3, NKX2-2, NKX2-5, NKX3-1, NKX6-1, NKX6-2, ONECUT1, ONECUT2, PAX2, PDX1, POU2F1, POU3F2, POU6F1, PRRX2, SRY, STAT4, STAT5A, TBP, TCF7, TCF7L2, TEF, ZNF384*
Heritable High Methylation	*AHR, ARID5B, ARNT, ASCL1, ATF1, ATF2, ATF3, ATF4, ATF7, BACH1, BACH2, BHLHB2, BHLHB3, CREB1, CREM, E2F1, E2F2, E2F3, E2F4, E2F5, E2F7, EGR1, EGR2, EGR3, EGR4, ELF1, ELF2, ELK1, ELK4, EP300, ERF, ERG, ESR1, ESR2, ETS1, ETS2, ETV7, FLI1, GCM1, GTF2I, HAND1, HAND2, HES1, HIF1A, HOXA5, KLF12, LMO2, MAF, MAFB, MAFF, MAFG, MAFK, MAX, MAZ, MEIS1, MITF, MTF1, MXD1, MXD4, MXI1, MYB, MYC, MYCN, MYF5, MYF6, MYOD1, MYOG, MZF1, NFE2, NFE2L1, NFE2L2, NFE2L3, NFIA, NFIC, NHLH1, NKX2-1, NR1H2, NR1H3, NR1I2, NR1I3, NR2F1, NR2F2, NR3C1, PATZ1, PAX5, PAX6, PGR, PPARA, RARA, RARB, RARG, REST, RUNX1, RUNX2, RUNX3, RXRA, RXRB, RXRG, SMAD1, SMAD2, SMAD3, SMAD4, SMAD5, SMAD6, SMAD7, SMAD9, SP1, SP2, SP3, SP4, SREBF1, SREBF2, TAL1, TAL2, TBX5, TCF12, TCF3, TCF4, TFAP2A, TFAP2B, TFAP2C, TFAP4, TFCP2, TFDP1, TFE3, TFEB, THRA, THRB, TLX2, UBP1, USF1, USF2, VDR, YY1, ZBTB6, ZEB1, ZIC1, ZIC2, ZIC3, ZNF148*

CGI microarray probe sequences were assessed for 459 human TFBS motifs from TRANSFAC using TFBS motif searching algorithm MATCH, with a significant individual TFBS enrichment threshold determined by Fisher's exact test and a p-value justified by Bonferroni correction of 0.05/459/5 = 2.2×10−5 (i.e., p = 0.05/459 TFBS motifs/five methylation maintenance categories) when background sets were selected as #1 in Table 2.

### Evaluation of regulators of DNA methylation that might contribute to “random” methylation

While the predominant finding of this study was that *cis*-acting DNA regulatory elements associate with non-random heritable DNA methylation patterns, we also identified a substantial number of loci showing no generational trends of DNA methylation inheritance, in noncompliance with the stochastic methylation maintenance model of Riggs *et al.* [11], [12], [44]. While extensive examination of mechanisms of such methylation “randomness” was beyond the scope of this study, we nonetheless identified various factors that could likely contribute to this (seemingly) disordered methylation patterning.

We assessed the expression of a number of genes encoding chromatin-modifying proteins that could potentially facilitate “random” DNA methylation (Table 4). Alterations in the expression of chromatin modifiers that might decrease DNA methylation included downregulation of genes encoding histone acetyltransferases (*MYST3* and *MYST4*), while gene expression changes that might increase random DNA methylation [24], [53] included downregulation of the DNA-methylation “insulator” gene, *CTCF*, upregulation of the *de novo* DNA methyltransferase *DNMT3B* gene, and upregulation of the CTCF-antagonist gene *CTCFL* (*BORIS*) [38], [54], [55]. Random *de novo* DNA methylation also occurs via DNMT recruitment to DNA repair complexes at sites of single- or double-strand breaks, which can result from DNA stand crosslinking by platinum drugs such as cisplatin (as used in this study) [29], [31], [56]. Taken together, we observed greater gene expression dysregulation that would tend to associate with increased, *cis*-regulatory element-independent (and perhaps, DNA replication-independent) DNA methylation, as compared to gene expression alterations facilitating decreased DNA methylation, although more extensive laboratory validation is required to substantiate this conjecture.

**Table 4:** Expression of genes encoding chromatin-modifying proteins that could potentially contribute to the “random” DNA methylation.

Chromatin-Modifying Genes	Fold-Change of Generation-5 Cells vs. Parental Cells (log2)	Hypothesized Function	Possible influence on DNA methylation	Reference(s)
*CTCF*	−1.28	methylation protection	increase	[75]
*CTCFL (BORIS)*	−1.54	Inhibition of methylation protection	decrease	[38]
*CXXC6 (TET1)*	1.42	hydroxylation of methylcytosine	likely decrease	[76]
*HMGB1*	1.26	repressive chromatin remodeling	increase	[77]
*DNMT3B*	1.80	de novo DNA methylation	increase	[78]
*MYST3*	−1.20	histone acetylation	increase	[79]
*MYST4*	−1.23	histone acetylation	increase	[79]

## Discussion

In this study, we used a microarray approach [34], [35], [57] for examining DNA methylation inheritance over five generations of the chemotherapy-sensitive ovarian cancer cell line A2780 [46], hypothesizing that some DNA-methylated loci might demonstrate reproducible DNA methylation heritability, despite the presence of various phenomena that might oppose such reproducibility. While the stochastic DNA methylation model allows for the propagation of methylated sequences with very high accuracy (as we observed in our highly and lowly heritably methylated CGI probe loci), it also provides for dynamic fluctuations that may, over time, result in the partial or complete loss of DNA methylation [11], [12]. In their seminal publication, Riggs *et al.* computationally predicted that from a cell population having an average of 50% methylation at any specific CpG dyad, the selection of two clones having 0% and 100% DNA methylation levels would, after approximately 30 generations, equilibrate to a steady state of 50% methylation at that particular dyad; that computational prediction was in large agreement with independent, experimental validation studies [6], [43], [52], [58]. As our cell generational studies were initiated with a single clone of the parental A2780 cells, we assert that specific CpG loci could likewise demonstrate progressive increases or decreases in DNA methylation, with an eventual equilibrium after 30 or more cell generations. As we examined DNA methylation after only the 1^st^, 3^rd^, and 5^th^ generations, such progressive methylation increases or decreases should still be occurring. Thus, for specific subpopulations of loci possessing high or low methylation levels and derived from a heterogeneously methylated cell population, our data is quite consistent with the stochastic model [11], [12].

The Riggs' stochastic model further predicts that starting from a mixed population, the propagation of CGI probe loci categorized as stochastic hypomethylation could exhibit an increase in failed methylation maintenance and/or an inadequate low rate of *de novo* methylation, while stochastically hypermethylated loci, also within an initially clonal population, might possess an increased rate of *de novo* methylation [20], [32], perhaps mediated by transcription factor recruitment of epigenetically repressive complexes to target genes [26]. In addition, our data suggests that distinct groups of TFs associate with high (*e.g.*, myogenic TFs) or low (*POUF* TF family genes) strictly heritable CGI probe loci, and also with stochastically hypermethylated (*FOX* forkhead TF genes) and stochastically hypomethylated (*TFAP* family TF genes) CGI probe loci (Table 3). Interestingly, we also observed greater hypomethylation, in the later generations, than in the parental cells, consistent with a recent DNA methylation analysis of platinum-resistant ovarian cancer [41]. At this juncture, however, we cannot ascribe any functional interpretation to specific TF gene associations with CGI probe loci displaying the four distinct modes of DNA methylation maintenance.

While we did observe enhanced fidelity of DNA methylation maintenance of CGIs enriched with TFBSs, we also noted substantial “random” methylation of non-TFBS-enriched loci (Figure 4 and Table 2). Although possibly related due to cisplatin exposure-related DNA damage, we hypothesize that such “randomly” heritably methylated loci were likely mostly unassociated with cisplatin resistance, based on our definition (Table 1) of random methylated loci as those exhibiting inconsistent methylation changes from generations 1 to 3 to 5. By contrast, CGI probe methylation levels contributing to drug resistance (following drug exposure) would likely, of necessity, require accurate preservation of those methylation levels in subsequent cell generations (to allow continued resistance) [59], [60]. While likely unrelated to cisplatin resistance, we speculate that such seemingly random methylation could have multiple bases. One previous study demonstrated that TFBS-unassociated CGIs recruit the Polycomb repressive complex-2 (PRC2) [61], which trimethylates lysine 27 of histone H3 (H3K27me3), representing a transcription-silencing histone “mark” that often precedes DNA methylation. Others have demonstrated that genes often DNA-methylated in tumors or during normal aging are frequently development (tissue lineage)-associated genes that copossess “bivalent” activating and repressive histone marks in embryonic stem (ES) cells [62]. We also observed changes in the expression of genes encoding various chromatin modifiers (Table 4), including expression alterations often correlated with decreased methylation, including upregulation of histone acetyltransferase genes (*MYST3*, *MYST4*). Gene expression changes that might correlate with increased DNA methylation included downregulation of the “insulator” protein-encoding gene *CTCF*, and upregulation of *DNMT3B* (encoding a *de novo* DNA methyltransferase), and the CTCF antagonist gene *BORIS* [24], [38], [55] (Table 4). Additionally as described above, cisplatin-associated DNA damage, upon subsequent DNA replication, can result in single- or double-strand breaks, which are now well associated with DNMT recruitment and *de novo* DNA methylation [29], [31], [46].

Based on the strong association of CGIs with *cis*-acting regulatory elements [1], [13], [15], we hypothesized that the methylation levels of CGIs possessing such elements might, of necessity, be propagated in a precise manner. Consequently, we examined the enrichment of TFBSs within CGI probe loci demonstrating the five distinct types of methylation maintenance (Table 1), as compared to randomly generated loci or genome-wide microarray probes lacking TFBSs. We found that compared to random sequences, TFBS motifs were well enriched within CGIs comprising the four methylation maintenance categories, in consistency with the stochastic methylation model. These results are in strong agreement with recent studies showing that CGI evolutionary conservation is often associated with DNA sequence (but not GC-content) [63], representing a possible future study by our group (*i.e.*, the identification of sequence motifs conferring DNA methylation fidelity).

As compared to heritably strict or stochastic DNA methylation, however, randomly propagated CGIs (*i.e.*, loci whose methylation propagation was inconsistent with the stochastic model) were completely devoid of enriched TFBSs (Tables 2). Although our objective was not to quantify the extent of methylation variance of specific probes categorized as random, we nevertheless noted a large degree of variation between generations 1 to 3 that was to some extent, reversed in generation 5 (Figure 4). We assert that this variance was not due to technical fluctuations, a variable well corrected for by the widely used Loess normalization [64], in addition to our own empirically based Bayesian algorithm [39]. Although it is difficult to hypothesize a biological mechanism for this finding, one might speculate that early chemotherapy selection of innately resistant clones (*e.g.*, between generations 1 and 3) might require greater methylome and transcriptome divergence, possibly required for the gain of tumor aggression, as compared to (minimally aggressive) the parental (drug-naive) cells [65].

These results also agree with a previous study, of normal epithelial cells, demonstrating that the methylation pattern error rate (MPER) in promoter-associated unmethylated CGIs, was over two-fold lower than the MPER of CGIs not associated with promoter regions [10]. A similarly decreased DNA methylation fidelity of promoter-dissociated CGIs was revealed in a study of monozygotic and dizygotic twins [66], while the loss of a −1.8 kb *cis*-regulatory element upstream of the *GATA2* gene resulted in complete loss of the maintenance of hypomethylation [67]. These studies, and our current results, might suggest some biological mechanism(s), yet to be identified, that preferentially enforces the accurate fidelity of methylation levels of CGIs proximal to regulatory elements, even in cancer cells (which are well known to possess decreased fidelity) [5], and also in cells having genomic instability or DNA damage (which is associated with random *de novo* methylation) [29], [31].

In summary, we have demonstrated non-random, heritably strict or stochastic propagation of DNA methylation levels within sequences possessing *cis*-regulatory elements, coexistent with considerable random methylation of sequences lacking such regulatory elements. These results support recent models of stochastic and strict heritable DNA methylation [9], [11], [12], [44], while also allowing for numerous other factors that could facilitate transient methylation states [1], [13], [15], [61]. Further studies to identify the existence of a possible biological mechanism(s) underlying enhanced fidelity of methylation levels in CGIs possessing *cis-*regulatory elements could provide insight into how DNA methylation patterns are maintained, and how dysregulation of this process could result in the inappropriate gene expression characteristic of numerous disease pathologies.

## Materials and Methods

### Cell culture studies

Cell culture reagents were obtained from Invitrogen Gibco/BRL (Carlsbad, CA). For these studies, we utilized our previously described cell culture model system for assessing the effects of a DNA damaging agent [47]. For these studies, we used the cisplatin-sensitive, epithelial ovarian cancer cell line A2780 (ATCC, Manassas, VA). Starting with a single clone, drug-sensitive parental A2780 cells were progressively exposed to increasing doses of the DNA crosslinking agent, cisplatin [47], over the course of five cell generations, using the cisplatin GI_70_ dose (*i.e.*, drug dose eliciting 70% growth inhibition, as determined by “MTT dose response curves,” followed by normalization to untreated cells, log transformation, and nonlinear curve fitting, using Prism 4.0 (GraphPad, La Jolla, CA)) for each specific generation, based on similar previous cisplatin DNA damage studies [46], [47]. Cisplatin treatments were administered for three hours, followed by a three-day recovery period, as we have described previously [47], [68], [69].

### Quantitative DNA methylation microarray assessments by differential methylation hybridization

Genomic DNA from generation-1, 3, and 5 cells was isolated using DNeasy DNA purification kits (Qiagen, Valencia, CA). To assess the fidelity of transgenerational DNA methylation heritability, in the presence of possible confounding factors (such as DNA damage, a cancer phenotype, etc.), we used a two-color array strategy [42] previously known as differential methylation hybridization [34], [35], [57], [69]. Briefly, isolated DNA was digested with the methylation-insensitive restriction enzyme BfaI (C∧TAG), followed by ligation of linkers. Linker-ligated DNA was then digested by the methylation-sensitive enzymes HinP1I (G∧CGC) and HpaII (C∧CGG), and the digestion products amplified by linker PCR. The PCR products were further amplified using aminoallyl-dUTP incorporation labeling of the methylation-dependent restricted DNA with the fluorophores Cy3 (green, parental A2780) or Cy5 (red, A2780 cells after 1, 3, and 5 cell generations [34], [35], [47], [57], [69]. The labeled DNA samples were then combined and hybridized to a customized 60-mer oligonucleotide microarray (Agilent, Santa Clara, CA) containing over 40,000 CpG-rich fragments from 12,000 known gene promoters. Following hybridization and washing, microarrays were scanned and images generated using an Axon GenePix 4200A scanner (Molecular Devices, Sunnyvale, CA), with data analysis described below. In addition, experiments were repeated with dyes swapped for parental *vs.* generation 3 cells.

### Differential methylation hybridization data analysis and definitions of DNA methylation heritability patterns

Raw DMH microarray data (GenePix GPR files) were first subjected to LOESS normalization, a method that allows correction for background and various microarray technological fluctuations (*e.g.*, differences in dye intensity related to spatial location and spot intensities), rather than biological differences [64]. An empirical Bayesian algorithm was then used to model the differentially methylated probes between the parental A2780 cells (Cy3) and generation 1, 3, and 5 cells (Cy5). This model incorporates both inter-sample variation (*i.e.* variation among three control samples) and intra-sample variations (*i.e.*, probe-specific pixel variations in foreground and background signals) [39], with all random variables modeled by normal distributions. Differentially methylated probes were determined by their empirical Bayesian probabilities, using a probability threshold of 0.80. All DMH DNA methylation data has been deposited, in MIAME format, in the Gene Expression Omnibus (http://www.ncbi.nlm.nih.gov/projects/geo/; accessible using SuperSeries code GSE15709).

By comparing the parental A2780 with their 1^st^, 3^rd^, and 5^th^ generation progeny cells, CGI microarray probes having increased methylation (probability≥0.80 by the empirical Bayesian algorithm) were defined as hypermethylated, while probes with decreased methylation (probability≥0.80) were defined as hypomethylated; otherwise, the methylation status of CGI probes was considered unchanged. As described in Table 1, our results yielded four possible patterns of transgenerational maintenance of DNA methylation, in accord with the stochastic model of Riggs *et al.*, which allows for highly or lowly methylated loci to ultimately achieve similar steady states of DNA methylation, due to fluctuations in the efficiency of methylation maintenance and/or *de novo* DNA methyltransferase activity [11], [12]. Consequently, CGI probes showing progressively increased methylation from generations 1 to 5 were defined as *stochastically hypermethylated*, while probes showing progressively decreased methylation were defined as *stochastically hypomethylated* (Table 1). Among CGI probes showing no change in methylation (probability <0.80 by the empirical Bayesian algorithm), those showing consistently high methylation (>85% of the maximal microarray fluorescence, regardless of generation, also having ≤15% changes between generations) were designated as *heritably highly methylated*. By contrast, CGI microarray consistently having <15% of the maximal fluorescence (regardless of cell generation, also having ≤15% changes between generations) were categorized as *heritably lowly methylated*. Both heritably high and low methylated loci were consistent with the faithful DNA maintenance mechanism of Riggs' model [11], [12]. Finally, probes demonstrating a mixture of increased and decreased methylation from generations 1 to 5 were assigned to a fifth category, *random methylation* (Table 1), a condition not described by the stochastic methylation maintenance model of Riggs *et al*. [11], [12].

### Analysis of the behavior of adjacent CGI probe loci

As adjacent probes, we chose those probes located in the same genes and within a distance of 1500 bp as being adjacent, as more than 90% of CGIs have length less than 1500 bp [70]. Thus, all the probes were clustered into a number of different clusters, with probes in the same cluster are adjacent to each other and probes in different clusters are not adjacent. As a result, probes from the same CGI are resided in the same cluster. If all the probes localized in the same cluster fall into the same category, the CGI represented by that cluster is in a consistent methylation heritability status; otherwise, the CGI should has multiple statuses.

### Gene expression microarrays

For gene expression analyses, total RNA was isolated from parental and fifth generation A2780 cells using RNeasy purification kits (Qiagen, Valencia, CA), reverse transcribed to cDNA, followed by *in vitro* transcription to biotinylated cRNA, purification, and labeling for hybridization, using our previously described method [47], [57]. All microarray hybridizations were performed in quadruplicate for each A2780 cell generation using Human U133 plus 2.0 GeneChips (Affymetrix, Santa Clara, CA), and is deposited in NCBI Gene Expression Omnibus (GEO, http://www.ncbi.nlm.nih.gov/projects/geo/), accessible through GEO SuperSeries GSE15709).

In addition to the assessment of propagation of DNA methylation patterns, another objective was to determine time-dependent and cell division-dependent changes in gene expression, using microarray profiling (Affymetrix) of fifth-generation total cellular mRNA, as compared to total RNA derived from the parental cell line. Using Bioconductor [71], present (P), absent (A) or marginal (M) calls were determined using an Affymetrix Micro Array Suite 5.0 (MS 5.0) algorithm [72]. Fraction presence, defined as the average present/absent (P/A) detection call (scores assigned as P = 1, M = 0.5 and A = 0) for the experimental or control groups, was calculated for each microarray probe, and probes with at least one group having a fraction presence of 0.5 were selected for future use. Welch's t-tests were determined for each probe using its log-transformed signal, with p-values<0.01 considered significant. To further support the statistical significance of probes having p<0.01, we applied a moderately stringent fold-change cutoff of ≥1.2 or ≤−1.2 for downregulation (thus allowing an acceptable balance between false discovery and false negative rates) [73], in addition to the p-value cutoffs, to determine genes showing significant expression changes.

Subsequent to the categorization of CGI probes into the five methylation maintenance categories (described above and in Table 1), to determine possible causes of our observed of “random” methylation, we examined the expression of various chromatin-regulating genes, in fifth *vs.* parental generation cells.

### Analysis for enrichment of transcription factor binding sites (TFBSs) within CpG islands (CGIs)

We used our previously reported method [17] to identify TFBSs within the microarray (Agilent) CGI probe sequences included in any of the five methylation maintenance categories. TFBS searching was performed using MATCH algorithm (http://www.bioinfo.de/isb/gcb01/poster/goessling.html) [58], a weight matrix-based software. Two background sequence sets were selected, with the first background sequence set containing randomly generated promoter sequences matched (to the categorized probes) for equal length and GC content, while the second background probe set was generated by random selection of all CGI microarray CGI probe sequences not falling into any of the five methylation categories (*i.e*, not meeting any of the methylation pattern criteria shown in Table 1), but retaining the same GC content and sequence length. The frequencies of the predicted TFBSs for each probe within the five methylation maintenance categories (Table 1), and background sequences, were then compared by Fisher's exact test. Bonferroni justification was implemented to justify 459 human transcription factor motifs, *i.e.*, an individual p-value threshold was chosen as 0.05/459/5 = 2.2×10^−5^, for multiple comparisons [74].

## References

[1] Allis CD, Jenuwein T, Reinberg D (2007). Epigenetics.

[2] Esteller M (2009). Epigenetics in biology and medicine.

[3] Barrero MJ, Boue S, Izpisua Belmonte JC (2010). Epigenetic mechanisms that regulate cell identity.. Cell Stem Cell.

[4] Mohammad HP, Baylin SB (2010). Linking cell signaling and the epigenetic machinery.. Nat Biotechnol.

[5] Ushijima T, Watanabe N, Shimizu K, Miyamoto K, Sugimura T (2005). Decreased fidelity in replicating CpG methylation patterns in cancer cells.. Cancer research.

[6] Jeong S, Liang G, Sharma S, Lin JC, Choi SH (2009). Selective anchoring of DNA methyltransferases 3A and 3B to nucleosomes containing methylated DNA.. Mol Cell Biol.

[7] Schermelleh L, Haemmer A, Spada F, Rosing N, Meilinger D (2007). Dynamics of Dnmt1 interaction with the replication machinery and its role in postreplicative maintenance of DNA methylation.. Nucleic Acids Res.

[8] Ooi SK, Bestor TH (2008). Cytosine methylation: remaining faithful.. Curr Biol.

[9] Goyal R, Reinhardt R, Jeltsch A (2006). Accuracy of DNA methylation pattern preservation by the Dnmt1 methyltransferase.. Nucleic Acids Res.

[10] Ushijima T, Watanabe N, Okochi E, Kaneda A, Sugimura T (2003). Fidelity of the methylation pattern and its variation in the genome.. Genome research.

[11] Riggs AD, Xiong Z (2004). Methylation and epigenetic fidelity.. Proc Natl Acad Sci U S A.

[12] Chen ZX, Riggs AD (2005). Maintenance and regulation of DNA methylation patterns in mammals.. Biochemistry and cell biology  =  Biochimie et biologie cellulaire.

[13] Illingworth RS, Bird AP (2009). CpG islands–‘a rough guide’.. FEBS letters.

[14] Larsen F, Gundersen G, Lopez R, Prydz H (1992). CpG islands as gene markers in the human genome.. Genomics.

[15] Deaton AM, Bird A (2011). CpG islands and the regulation of transcription.. Genes & development.

[16] Voo KS, Carlone DL, Jacobsen BM, Flodin A, Skalnik DG (2000). Cloning of a mammalian transcriptional activator that binds unmethylated CpG motifs and shares a CXXC domain with DNA methyltransferase, human trithorax, and methyl-CpG binding domain protein 1.. Molecular and cellular biology.

[17] Li L, Cheng AS, Jin VX, Paik HH, Fan M (2006). A mixture model-based discriminate analysis for identifying ordered transcription factor binding site pairs in gene promoters directly regulated by estrogen receptor-alpha.. Bioinformatics.

[18] Li M, Paik HI, Balch C, Kim Y, Li L (2008). Enriched transcription factor binding sites in hypermethylated gene promoters in drug resistant cancer cells.. Bioinformatics.

[19] Choy MK, Movassagh M, Goh HG, Bennett MR, Down TA (2010). Genome-wide conserved consensus transcription factor binding motifs are hyper-methylated.. BMC Genomics.

[20] Gebhard C, Benner C, Ehrich M, Schwarzfischer L, Schilling E (2010). General transcription factor binding at CpG islands in normal cells correlates with resistance to de novo DNA methylation in cancer cells.. Cancer Res.

[21] Stirzaker C, Song JZ, Davidson B, Clark SJ (2004). Transcriptional gene silencing promotes DNA hypermethylation through a sequential change in chromatin modifications in cancer cells.. Cancer research.

[22] Thomson JP, Skene PJ, Selfridge J, Clouaire T, Guy J (2010). CpG islands influence chromatin structure via the CpG-binding protein Cfp1.. Nature.

[23] Zaidi SK, Young DW, Montecino M, van Wijnen AJ, Stein JL (2011). Bookmarking the genome: maintenance of epigenetic information.. The Journal of biological chemistry.

[24] Valenzuela L, Kamakaka RT (2006). Chromatin insulators.. Annual review of genetics.

[25] Leu YW, Yan PS, Fan M, Jin VX, Liu JC (2004). Loss of estrogen receptor signaling triggers epigenetic silencing of downstream targets in breast cancer.. Cancer Res.

[26] Hervouet E, Vallette FM, Cartron PF (2009). Dnmt3/transcription factor interactions as crucial players in targeted DNA methylation.. Epigenetics : official journal of the DNA Methylation Society.

[27] Robertson KD (2005). DNA methylation and human disease.. Nature reviews Genetics.

[28] Damiani LA, Yingling CM, Leng S, Romo PE, Nakamura J (2008). Carcinogen-induced gene promoter hypermethylation is mediated by DNMT1 and causal for transformation of immortalized bronchial epithelial cells.. Cancer research.

[29] Mortusewicz O, Schermelleh L, Walter J, Cardoso MC, Leonhardt H (2005). Recruitment of DNA methyltransferase I to DNA repair sites.. Proceedings of the National Academy of Sciences of the United States of America.

[30] Tost J (2008). Epigenetics.

[31] Ha K, Lee GE, Palii SS, Brown KD, Takeda Y (2011). Rapid and transient recruitment of DNMT1 to DNA double-strand breaks is mediated by its interaction with multiple components of the DNA damage response machinery.. Human molecular genetics.

[32] Ting AH, McGarvey KM, Baylin SB (2006). The cancer epigenome–components and functional correlates.. Genes Dev.

[33] Chen T, Hevi S, Gay F, Tsujimoto N, He T (2007). Complete inactivation of DNMT1 leads to mitotic catastrophe in human cancer cells.. Nature genetics.

[34] Yan PS, Wei SH, Huang TH (2002). Differential methylation hybridization using CpG island arrays.. Methods in molecular biology.

[35] Ahluwalia A, Yan P, Hurteau JA, Bigsby RM, Jung SH (2001). DNA methylation and ovarian cancer. I. Analysis of CpG island hypermethylation in human ovarian cancer using differential methylation hybridization.. Gynecologic oncology.

[36] Esteller M (2007). Cancer epigenomics: DNA methylomes and histone-modification maps.. Nat Rev Genet.

[37] Jones PA, Baylin SB (2007). The epigenomics of cancer.. Cell.

[38] Linhart HG, Lin H, Yamada Y, Moran E, Steine EJ (2007). Dnmt3b promotes tumorigenesis in vivo by gene-specific de novo methylation and transcriptional silencing.. Genes Dev.

[39] Jeong J, Li L, Liu Y, Nephew KP, Huang TH (2010). An empirical Bayes model for gene expression and methylation profiles in antiestrogen resistant breast cancer.. BMC medical genomics.

[40] Ehrlich M (2009). DNA hypomethylation in cancer cells.. Epigenomics.

[41] Yu W, Jin C, Lou X, Han X, Li L (2011). Global analysis of DNA methylation by methyl-capture sequencing reveals epigenetic control of Cisplatin resistance in ovarian cancer cell.. PLoS ONE.

[42] Dabney AR, Storey JD (2007). A new approach to intensity-dependent normalization of two-channel microarrays.. Biostatistics.

[43] Liang G, Chan MF, Tomigahara Y, Tsai YC, Gonzales FA (2002). Cooperativity between DNA methyltransferases in the maintenance methylation of repetitive elements.. Mol Cell Biol.

[44] Jones PA, Liang G (2009). Rethinking how DNA methylation patterns are maintained.. Nat Rev Genet.

[45] Xie H, Wang M, de Andrade A, Bonaldo MD, Galat V (2011). Genome-wide quantitative assessment of variation in DNA methylation patterns.. Nucleic acids research.

[46] Li J, Wood WH, Becker KG, Weeraratna AT, Morin PJ (2007). Gene expression response to cisplatin treatment in drug-sensitive and drug-resistant ovarian cancer cells.. Oncogene.

[47] Li M, Balch C, Montgomery JS, Jeong M, Chung JH (2009). Integrated analysis of DNA methylation and gene expression reveals specific signaling pathways associated with platinum resistance in ovarian cancer.. BMC Med Genomics.

[48] Estecio MR, Issa JP (2011). Dissecting DNA hypermethylation in cancer.. FEBS letters.

[49] Landolin JM, Johnson DS, Trinklein ND, Aldred SF, Medina C (2010). Sequence features that drive human promoter function and tissue specificity.. Genome research.

[50] Ramirez-Carrozzi VR, Braas D, Bhatt DM, Cheng CS, Hong C (2009). A unifying model for the selective regulation of inducible transcription by CpG islands and nucleosome remodeling.. Cell.

[51] Pfeifer GP, Steigerwald SD, Hansen RS, Gartler SM, Riggs AD (1990). Polymerase chain reaction-aided genomic sequencing of an X chromosome-linked CpG island: methylation patterns suggest clonal inheritance, CpG site autonomy, and an explanation of activity state stability.. Proc Natl Acad Sci U S A.

[52] Laird CD, Pleasant ND, Clark AD, Sneeden JL, Hassan KM (2004). Hairpin-bisulfite PCR: assessing epigenetic methylation patterns on complementary strands of individual DNA molecules.. Proc Natl Acad Sci U S A.

[53] Pillus L (2008). MYSTs mark chromatin for chromosomal functions.. Current opinion in cell biology.

[54] Grasser KD, Launholt D, Grasser M (2007). High mobility group proteins of the plant HMGB family: dynamic chromatin modulators.. Biochimica et biophysica acta.

[55] Klenova EM, Morse HC, Ohlsson R, Lobanenkov VV (2002). The novel BORIS + CTCF gene family is uniquely involved in the epigenetics of normal biology and cancer.. Seminars in cancer biology.

[56] Sorenson CM, Eastman A (1988). Mechanism of cis-diamminedichloroplatinum(II)-induced cytotoxicity: role of G2 arrest and DNA double-strand breaks.. Cancer Res.

[57] Fan M, Yan PS, Hartman-Frey C, Chen L, Paik H (2006). Diverse gene expression and DNA methylation profiles correlate with differential adaptation of breast cancer cells to the antiestrogens tamoxifen and fulvestrant.. Cancer research.

[58] Kel AE, Gossling E, Reuter I, Cheremushkin E, Kel-Margoulis OV (2003). MATCH: A tool for searching transcription factor binding sites in DNA sequences.. Nucleic Acids Res.

[59] Shen L, Kondo Y, Ahmed S, Boumber Y, Konishi K (2007). Drug sensitivity prediction by CpG island methylation profile in the NCI-60 cancer cell line panel.. Cancer Res.

[60] Baylin SB (2011). Resistance, epigenetics and the cancer ecosystem.. Nat Med.

[61] Mendenhall EM, Koche RP, Truong T, Zhou VW, Issac B (2010). GC-rich sequence elements recruit PRC2 in mammalian ES cells.. PLoS genetics.

[62] Rakyan VK, Down TA, Maslau S, Andrew T, Yang TP (2010). Human aging-associated DNA hypermethylation occurs preferentially at bivalent chromatin domains.. Genome research.

[63] Cohen NM, Kenigsberg E, Tanay A (2011). Primate CpG islands are maintained by heterogeneous evolutionary regimes involving minimal selection.. Cell.

[64] Smyth GK, Speed T (2003). Normalization of cDNA microarray data.. Methods.

[65] Glasspool RM, Teodoridis JM, Brown R (2006). Epigenetics as a mechanism driving polygenic clinical drug resistance.. Br J Cancer.

[66] Kaminsky ZA, Tang T, Wang SC, Ptak C, Oh GH (2009). DNA methylation profiles in monozygotic and dizygotic twins.. Nature genetics.

[67] Snow JW, Trowbridge JJ, Fujiwara T, Emambokus NE, Grass JA (2010). A single cis element maintains repression of the key developmental regulator Gata2.. PLoS genetics 6.

[68] Abbosh PH, Montgomery JS, Starkey JA, Novotny M, Zuhowski EG (2006). Dominant-negative histone H3 lysine 27 mutant derepresses silenced tumor suppressor genes and reverses the drug-resistant phenotype in cancer cells.. Cancer research.

[69] Balch C, Yan P, Craft T, Young S, Skalnik DG (2005). Antimitogenic and chemosensitizing effects of the methylation inhibitor zebularine in ovarian cancer.. Molecular cancer therapeutics.

[70] Gardiner-Garden M, Frommer M (1987). CpG islands in vertebrate genomes.. J Mol Biol.

[71] Gentleman RC, Carey VJ, Bates DM, Bolstad B, Dettling M (2004). Bioconductor: open software development for computational biology and bioinformatics.. Genome biology.

[72] Hubbell E, Liu WM, Mei R (2002). Robust estimators for expression analysis.. Bioinformatics.

[73] McClintick JN, Edenberg HJ (2006). Effects of filtering by Present call on analysis of microarray experiments.. BMC bioinformatics.

[74] Strassburger K, Bretz F (2008). Compatible simultaneous lower confidence bounds for the Holm procedure and other Bonferroni-based closed tests.. Statistics in medicine.

[75] Mukhopadhyay R, Yu W, Whitehead J, Xu J, Lezcano M (2004). The binding sites for the chromatin insulator protein CTCF map to DNA methylation-free domains genome-wide.. Genome Res.

[76] Wu SC, Zhang Y (2010). Active DNA demethylation: many roads lead to Rome.. Nat Rev Mol Cell Biol.

[77] Loukinov DI, Pugacheva E, Vatolin S, Pack SD, Moon H (2002). BORIS, a novel male germ-line-specific protein associated with epigenetic reprogramming events, shares the same 11-zinc-finger domain with CTCF, the insulator protein involved in reading imprinting marks in the soma.. Proc Natl Acad Sci U S A.

[78] Teschendorff AE, Menon U, Gentry-Maharaj A, Ramus SJ, Weisenberger DJ (2010). Age-dependent DNA methylation of genes that are suppressed in stem cells is a hallmark of cancer.. Genome Res.

[79] Grasser KD, Launholt D, Grasser M (2007). High mobility group proteins of the plant HMGB family: dynamic chromatin modulators.. Biochim Biophys Acta.

